# Cohort Profile Update: Manhiça Health and Demographic Surveillance System (HDSS) of the Manhiça Health Research Centre (CISM)

**DOI:** 10.1093/ije/dyaa218

**Published:** 2021-01-16

**Authors:** Ariel Nhacolo, Edgar Jamisse, Orvalho Augusto, Teodomiro Matsena, Aura Hunguana, Inácio Mandomando, Carlos Arnaldo, Khátia Munguambe, Eusébio Macete, Pedro Alonso, Francisco Saúte, Charfudin Sacoor

**Affiliations:** 1 Manhiça Health Research Center, Manhiça District, Mozambique; 2 National Institute of Health, Ministry of Health, Maputo, Mozambique; 3 Eduardo Mondlane University, Mozambique, Maputo, Mozambique; 4 National Directorate of Health, Ministry of Health, Maputo, Mozambique; 5 Barcelona Institute for Global Health, Barcelona, Spain

## The original cohort

The Manhiça Health Research Centre [Centro de Investigação em Saúde de Manhiça (CISM)] has been running a Health and Demographic Surveillance System (HDSS) since 1996, in the district of Manhiça, 80 km North of Maputo City (the capital of Mozambique). As indicated in the original cohort profile,^1^ the objective of this HDSS is to provide a platform for conducting biomedical research in local priority areas such as malaria, HIV/AIDS, tuberculosis (TB) and invasive bacterial diseases (IBDs), and to promote the training of young Mozambican scientists in biomedical, demographic, anthropological and other critical research areas.

## What is the reason for the new focus or new data collection?

**Figure 1:**
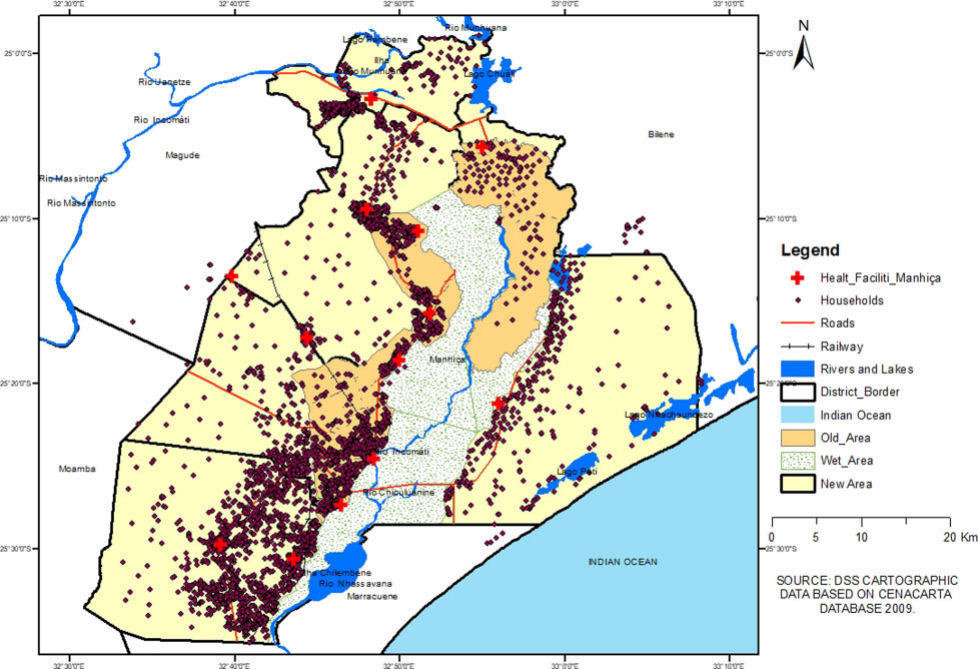
Map of Manhiça district showing the old and the new HDSS areas (Source: Created by the authors using HDSS cartographic data). CENACARTA, Centro Nacional de Cartografia e Teledetecção

After the publication of the original profile in 2013, CISM expanded the demographic surveillance area (DSA) in 2014, to cover the entire district in order to accommodate studies requiring lager sample sizes, and has experienced several changes. The expansion of the DSA is amongst the major improvements that CISM has been keen to achieve since its creation in 1996 in order to overlap the hospital catchment areas with that of the HDSS, but financial constraints have prevented it, particularly those related to sustainability of the HDSS in very remote areas. This non-overlap has also prevented comparisons of the HDSS data with those from the vital events registration system of the district; it also reduced the generalization of the results of some studies at the district level, e.g. studies that considered that the differences in variables such as population density and access to health care were important factors for generalizations. For instance, in a study on community HIV transmission it felt inappropriate to generalize the findings throughout the district because the old areas have higher population density than the new areas, which could cause differences in the rate of HIV transmission.^2^ Further, some studies, such as one on the effectiveness of pneumococcal vaccine, required a larger sample size that the old areas could not provide in the duration of the study. Figure 1 presents the old and the new DSS areas.

The data collection tool was changed from Personal Digital Assistants (PDAs) to tablets due to technical difficulties. Tablets were adopted also to accommodate the Open Health Demographic System (OpenHDS)—a paper-free software for HDSS. Most INDEPTH Network sites were happy to adopt this tool because it was developed specifically for HDSS by professionals who participated in the development of the paper-based tool called the Household Registration System (HRS), and had been involved in conceptual and technical issues of HDSSs within INDEPTH, including data manipulation and analysis.^3^

Simultaneously, the HDSS started to collect additional data because the original data had some limitations for demographic and socio-economic analysis, particularly in relation to the causes and/or determinants of health and demographic dynamics. Difficulties in editing HRS to accommodate new questions in a system of relational and longitudinal databases contributed to a delay in improvements—though three new modules (independent from the main relational database) were added, as indicated in,^1^ namely immunization, malaria control tools and household assets. So the transition to OpenHDS represented an opportunity to review and improve all the modules, resorting to Open Data Kit software if the questions were not in the OpenHDS core questionnaires (see section ‘What has been measured?’ for details). To update the DSS data, each household is visited at least twice per year to collect data on pregnancies, pregnancy outcomes, migration and other variables, at individual and household levels. Every Friday all the tablets are brought to CISM’s offices to transfer the data into the server, using Wi-Fi. A light copy of the database is transferred back into the tablets after synchronization.

In November 2019, the modules of the hospital-based morbidity surveillance (which is part of the HDSS) were improved to collect data on nutrition, diagnoses from malaria rapid tests, and the demographic characteristics of the person accompanying the child to the health facility. As indicated in^1^^,^^4^ CISM runs a 24-hours hospital-based morbidity surveillance for children <15 years old in 7 out of 15 health facilities in the district. DSS ID cards for these children are printed and distributed at home by the DSS fieldworkers. Each time that a child visits the outpatient or inpatient services, a specific form is filled-in with health data, clinical examinations, diagnosis, and treatment or referral undertaken. The form has a space to fill-in the DSS ID from the card, so that all the demographic and illness events are linked to each child by this ID. These forms are brought to CISM’s data centre, where they are double-entered in a specific database. Another improvement of this morbidity surveillance was the replacement of verbal informed consent by written informed consent, signed by the person accompanying the child to the health facility.

In relation to verbal autopsies, Manhiça adopted the World Health Organization (WHO) 2016 version of questionnaires in 2017, replacing the 2012 version. The data are collected using tablets for all ages, and the causes of death are assigned using *InterVA* software (See^5^ for details of this software).

**Figure 2:**
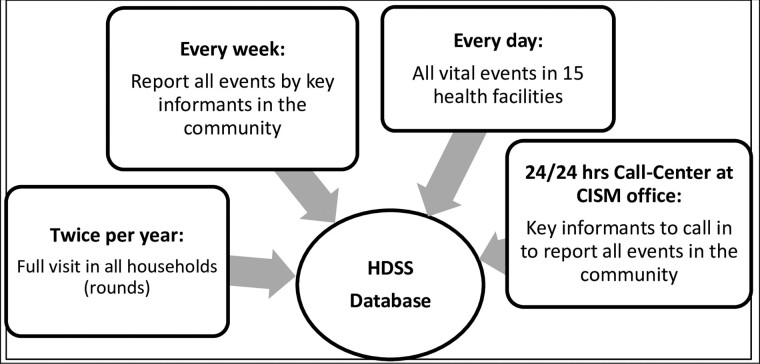
Diagram showing the four mechanisms for demographic data collection in Manhiça HDSS since 2017

In 2016, the HDSS established a free call centre for speeding up the reporting of demographic events. Manhiça is participating in the Child Health and Mortality Prevention Surveillance (CHAMPS) Network, a multi-centre study aiming to ascertain the causes of death in children <5 years old and stillbirths (see www.champshealth.org, for details). This study requires notification of child deaths and stillbirths within 24 h after occurrence, to investigate causes of deaths using minimal invasive tissue sampling of the dead bodies. This required innovative approaches in the HDSS surveillance and reporting scheme such as the call centre, which works 24 h a day, for community members, fieldworkers, medical staff and the police, to call in to report the events occurring in the community and health facilities. In addition, the number of key informants was increased and they were provided with mobile phones to ensure timely case notification. Figure 2, updated from the 2013 profile, includes this call centre in the mechanisms for updating demographic data in Manhiça. However, to avoid selection bias on reporting of other events such as pregnancies, live births, abortions, adult deaths and migration, the informants are instructed to report all these events as well (see^1^ for details on how CISM updates the HDSS data).

**Figure 3:**
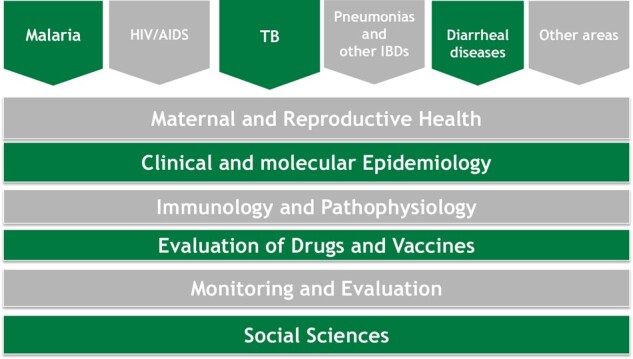
Research matrix of the Manhiça Health Research Center in 2019

CISM has started a new focus on malaria elimination in line with the new Technical Global Strategy of the WHO, which led to a massive investment in the Magude pilot project, including the establishment of a HDSS. Magude is a neighbouring district of Manhiça, in Maputo province, that was selected for the evaluation of a malaria-elimination project to inform the elimination strategy for the country’s Southern region.^6^ Also, related to this, CISM restarted entomological research with the establishment of specific infrastructure, including human resources and a fully-equipped and functional insectary. Another area of work that saw a remarkable spike was research on health systems, particularly on health information systems in the provinces of Maputo and Gaza, Southern Mozambique. CISM has also increased its scientific and geographical scope to include the testing of new interventions to prevent negative maternal outcomes due to eclampsia and pre-eclampsia in the provinces of Maputo and Gaza, and those due to malaria in the provinces of Nampula, Zambézia, and Sofala, as well as environmental health studies related to mineral resources in Cabo Delgado (see the CISM’s research matrix in Figure 3). This demonstrates the ability of CISM to expand and contract the platforms according to the demand of research activities, while maintaining a core study area in Manhiça.

CISM has also started working on neglected tropical diseases, particularly on soil-transmitted helminthiasis and schistosomiasis.

## What will be the new areas of research?

In Manhiça, there is a work underway to review and update the levels and trends of basic demographic indices, and the improvements made in the HDSS content will allow further analysis in top priority areas such as reproductive health among adolescents, household dynamics, orphanhood, early motherhood and fatherhood, marriages and divorces, causes of migration, population ageing, adult mortality, and causes of death—and how all these impact on the well-being of population and individuals in poor resource setting such as Manhiça.

In 2019, CISM has reviewed its strategic plan and the research areas, which will now include environmental health and non-communicable diseases in collaboration with other institutions.

## Who is in the cohort?

**Figure 4:**
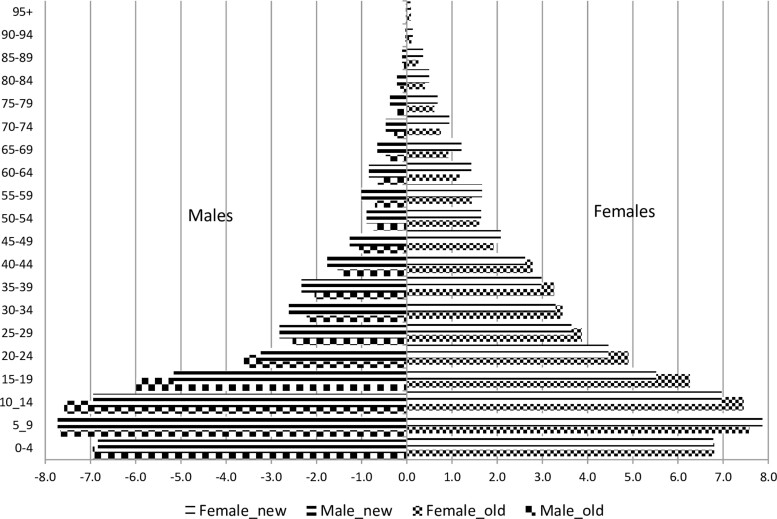
Age and sex composition of the population in Manhiça demographic surveillance area, 2019 (old and new areas). (Source: Manhiça HDSS databases). The *x*-axis presents the percentage of each age-group and sex relative to the total population. Negative percentages were used only for graphical purposes (to force Excel software to display males on the left side of the *y*-axis, the vertical line showing a percentage of 0.0). The *y* axis presents 5-years age-groups of population, in years. Male_new and male_old (or female_new and female_old) stand for male (or female) population in new areas and old areas, respectively

**Figure 5:**
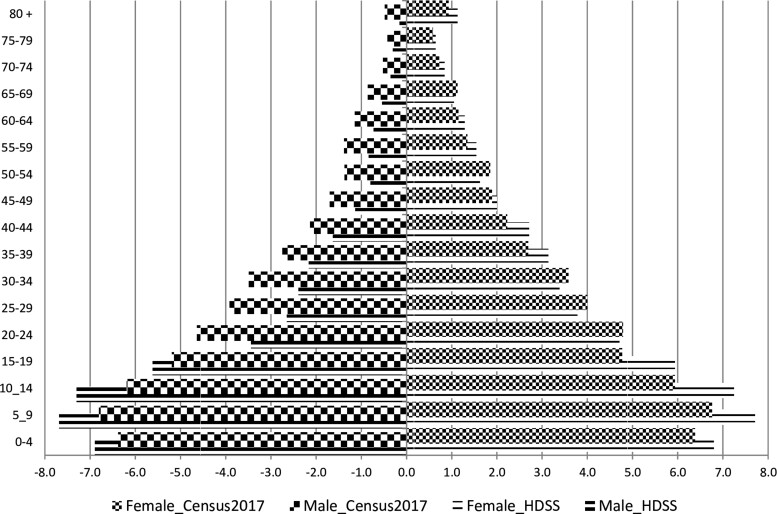
Age and sex composition of the population in Manhiça district according to the national census in 2017 and according to Manhiça HDSS databases in 2019. [Source: Manhiça HDSS databases; and INE (2019^8^)].The *x*-axis presents the percentage of each age-group and sex relative to the total population. Negative percentages were used only for graphical purposes (to force Excel software to display males on the left side of the *y*-axis, the vertical line showing a percentage of 0.0). The *y*- axis presents 5-years age-groups of population, in years. Male_HDSS and male_Census2017 (or female_HDSS and female_Census2017) stand for male (or female) population in Manhiça district according to Manhica HDSS and according to the national census conducted in 2017, respectively

The criteria for defining HDSS members has been the same since 1996^1^^,^^4^ and the baseline censuses during the expansions of the DSA followed the same methods as in the 1996 baseline census. Procedures for data cleaning have also remained the same, including those designed to avoid and correct duplicated individuals who are found in the surrounding neighbourhoods during expansions, after having emigrated from the old areas—a typical challenge in this geographically growing cohort. The 2014 expansion enumerated 72 494 individuals, which increased the study population from 97 496 inhabitants to a total of 169 990. Now, the HDSS covers 2380 km^2^.^7^ The most recent complete round of data collection terminated in December 2019, showing that in December 2019 the DSA had 201 845 inhabitants living in 46 441 households. Figure 4 presents the age and sex composition (population pyramid) of the populations in the old and in the new areas, and it shows that the two populations have similar structures although the old area has a higher imbalance between adult males and females than the new area. This male-to-female imbalance has been reported earlier and was attributed to higher migration and mortality among adult males than among females.^4^ However, this figure shows a decrease in the number of children aged 0–4 years compared with those aged 5–9 years—a new shape that was also captured by the 2017 National Census^8^ in the district of Manhiça, as indicated in Figure 5.

**Figure 6:**
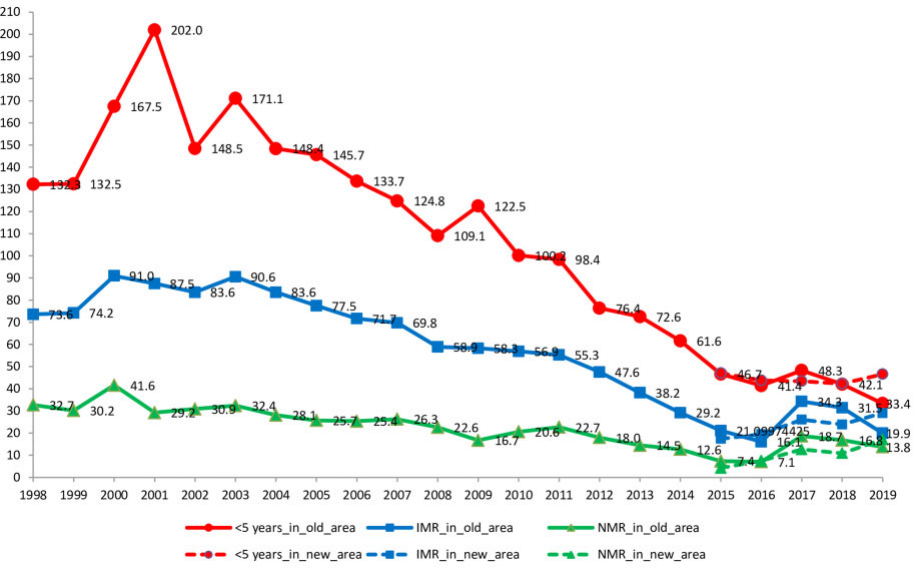
Mortality rates in neonates, infants and children <5 years old, Manhiça 1998–2019 by study area (old vs new areas). (Source: Manhiça HDSS database). The *x*-axis presents the calendar year to which the rate refers. The *y*-axis presents the annual rate of mortality per 1000 live births. The lines labelled <5 years_in_old_area and <5 years_in_new_area stand for the conventional mortality rate in children <5 years in old and new areas, respectively. The lines labelled IMR_in_old_area and IMR_in_new_area stand for infant mortality rate in old and new areas, respectively. The lines labelled NMR_in_old_area and NMR_in_new_area stand for neonatal mortality rate in old and new areas, respectively

Mortality in children <5 years old has continued to decrease in Manhiça, from 132 deaths in under-fives per 1000 live births in 1998 to 76.4 in 2013 and 46.6 in 2019 (Figure 6). This figure also shows some increase in mortality in under-fives during the past 5 years, after having reached a lower level in 2015. The new areas appear to have lower mortality than the old ones—somehow running in contrast with what would be expected if taking into account that the new areas are relatively poorer than the old areas.

## What has been measured?

As indicated earlier, in 2014 new questions were added, and old questions were rephrased or the answers were improved—e.g. the question on relationship to the head of household had 7 options in the HRS system, 9 in PDAs, but now it has 15. The following are the main categories of new variables added in the HDSS after the publication of the original profile in 2013.

At household level—socio-demographic characteristics of the head of household and whether he/she is a resident member, household assets, access to drinking water, sanitation (availability and hygiene of toilets), electricity, livestock, poultry and cellphone numbers.

At individual level—relationship to the main head and to the substitute head, literacy (‘can you read the sentence in this card?’), official documents and whether they have expired or not. Marital status and date of transition to the current status (where applicable), geographic place where the transition took place, place of birth and mother language (as a proxy for ethnicity). Motives for migration, relationship to the head at origin and destination household, religion, occupation, disability and cause of disability. When a member dies, place of death (hospital, home and geographic place of death). Pregnancy—gestational age, antenatal visits card, malaria intermittent preventive treatment (IPT), bed-nets and woman’s phone number. Fertility histories for women of reproductive age and orphanhood for children <15 years old.

## What has it found?

Table 1 presents the population and households in December 2019, by study area and by Administrative Post in Manhiça DSA (an administrative post is the level below a district in the administrative division of Mozambique; the districts are below the provinces—the largest administrative divisions). Population density is 84.8/km^2^ but it is higher in the old area (226.8/km^2^, where 56% of the population lives) than in the newly incorporated study area (47/km^2^), where some spaces are not inhabited either because they are coastal areas of the Indian Ocean or are the floodplains of the Incomati River. Most households in the old area are larger and headed by females (52.9 %) than in the new area. Sons or daughters of the heads and the heads themselves are the majority in these households, followed by the spouses and daughters-in-law (Table 2).

**Table 1:** Population and households by study area in the Manhia HDSS, 31 December 2019. (Source: Manhia HDSS databases)

Spatial distribution of population		Population characteristics			Households			
		Male	Female	Total	Number	%	% Female headed	Median size
DSS area	Old area (500 km^2^)	50 098	62 684	112 782	24 540	52.5	52.9	6
	New area (1880 km^2^)	40 018	48 557	88 575	22 186	47.5	48.9	5
Total	2380 km^2^	90 116	111 241	201 357	46 726	100.0	50.9	5
Administrative Post	3 de Fevereiro	20 974	27 812	48 786	10 472	24.4	61.6	6
	Calanga	3498	4256	7754	2003	4.3	50.7	5
	Ilha Josina Machel	3463	4825	8288	1844	3.9	62.0	6
	Maluana	12 548	15 270	27 818	7333	15,7	46.1	5
	Manhiça -Sede	35 652	42 827	78 479	17 801	38.1	48.0	6
	Xinavane	13 981	16 251	30 232	7273	15.6	45.4	5
Total		90 116	111 241	201 357	46 726	100.0	50.9	5

**Table 2:** Selected characteristics of households in Manhiça HDSS, 31 December 2019, by study area. (Source: Manhiça HDSS databases)

Household composition				Source of water (% households per study area)			
Relation to head	Old %	New %	All areas %	Water source	Old %	New %	All areas %
Son/daughter	41.0	38.5	39.9	Piped inside the yard	95.2	85.2	91.6
Head	21.9	26.2	23.8	Piped in the house	2.4	2.0	2.3
Grandson/ granddaughter	15.0	13.7	14.4	Protected dug well	0.1	0.5	0.3
Spouse	9.1	10.2	9.5	Unprotected dug well	0.5	3.8	1.7
Nephew	3.4	2.5	3.0	Hole with manual pump^a^	1.6	8.1	3.9
Daughter-in-law	2.5	2.2	2.4	Other	0.0	0.1	0.0
Brother/sister	1.8	1.4	1.6	Total of households	100.0	100.0	100.0
Stepson/stepdaughter	1.7	1.8	1.8	Has latrine (% households per study area)			
Other	1.3	1.1	1.2	Yes	96.0	77.4	87.2
Father/mother	0.8	0.8	0.8	No	4.0	22.6	12.8
Brother-in-law/sister-in-law	0.6	0.5	0.5	Total	100.0	100.0	100.0
Unrelated	0.6	0.8	0.7	Type of latrine, if any (% of households)			
Cousin	0.2	0.2	0.2	Ventilated toilet	71.0	65.5	68.7
Uncle/auntie	0.1	0.1	0.1	Flush toilet	20.6	17.0	19.1
Adopted	0.0	0.0	0.0	Traditional latrine	3.6	12.4	7.3
Total	100.0	100.0	100.0	Unventilated toilet	4.8	5.1	4.9
				Total	100	100	100

a Defined, in the HDSS manual, as a deep hole that has been drilled in order to reach underground water reserves. The water is supplied from a borehole or pipe through a pump.

A very high proportion of households have piped water in the dwelling unit, particularly in the old areas (95.2 %), but sanitation raises concerns, particularly in the new areas, where 22.6% of households have no toilet (Table 2).

As with mortality in children <5 years old, fertility has been declining in the study area, from a total fertility rate of 5 children per woman in the 1998–2000 to 4.4 in 2013, and to 3 in the most recent years. Details on the levels and trends of fertility, mortality and migration are in preparation for specific research articles for publication.

## What are the main strengths and weaknesses?

One of the strengths of Manhiça HDSS now is that covering the whole district can permit the estimation of the coverage and accuracy of the data collected by the vital event registration system in Manhiça, including comparisons and validation of demographic indices estimated from these and other sources such as National Censuses and other surveys at district level. In terms of human resources, the HDSS is managed by two demographers with MSc qualifications and two geographers with BSc qualifications—they have been crucial for the implementation of the research projects at CISM. Now the two MSc graduates are undergoing PhD training using Manhiça HDSS data, which will permit more publications on the demography and health of Mozambique, and will improve their support of the design and implementation of bio-medical and social research projects in Manhiça and other collaborating institutions, including improvements in their abilities to train, teach and supervise MSc students from Mozambique and other countries.

The weaknesses include the fact that OpenHDS is a new software and still needs improvement and customization for the local context, including those related to data cleaning. Although the DSS covers the whole district, the hospital-based morbidity surveillance is running in only 7 health facilities out of 15. Further, this morbidity surveillance does not include adults aged ≥15 years due to financial constraints, which represents a handicap for the study of the disease profile of Manhiça’s population.

## Ethics approval

The HDSS data collection has ethical approval from the Institutional Ethics Review Board for Health at CISM (approval no. CIBS_CISM/01/12), and from the National Bioethics Committee for Health (approval no. 174/CNBS/12).

## Can I get hold of the data? Where can I find out more?

The Manhiça HDSS data are not openly accessible at a web address, but as indicated in the original profile, they are shared within the INDEPTH Network. Please send formal requests for data to Godifre Capinga (godifre.capinga@manhica.net), accompanied by a proposal that will be analysed by CISM’s internal scientific and ethical committees.

**Profile in a nutshell**

- The Manhiça Health Research Centre (CISM) has been running a Health and Demographic Surveillance System (HDSS) since 1996, in Manhiça district, Mozambique.
- In 2014, the HDSS was extended to cover the entire district, which increased the population from 97 496 inhabitants to 169 990. Currently (December 2019), the HDSS area is 2380 km^2^, with 201 845 inhabitants and 46 441 households.
- Personal Digital Assistants were replaced by tablets and OpenHDS was introduced as the paper-free software for the HDSS. A call centre was established to speed up the notification of demographic events.
- The data were improved to permit more informative analyses of demographic dynamics, adding new or editing existing questions at household and individual levels.
- The research focus was widened to include malaria elimination, entomology, neglected tropical diseases and health systems in Manhiça and other areas of southern, central and northern Mozambique.
- To request these data please contact Godifre Capinga (godifre.capinga@manhica.net).

## Funding

The core funding for CISM’s activities comes from the Spanish Agency for International Development and Cooperation. The 2014 expansion of the DSA was funded by Global Alliance for Vaccines through the Institute for Health Metrics and Evaluation. The Bill and Melinda Gates Foundation through the Emory University is funding the maintenance of the DSS in these new areas.
